# Seroprevalence of Immunoglobulin E Antibodies against Japanese Cedar Pollen Allergens Cry j 1 and Cry j 2 in Dogs Bred in Japan

**DOI:** 10.3390/vetsci5030079

**Published:** 2018-09-11

**Authors:** Takashi Kuribayashi, Davide Cossu, Eiichi Momotani

**Affiliations:** 1 Laboratory of Immunology, School of Life and Environmental Science, Azabu University, Fuchinobe 1-17-71, Chuo-ku, Sagamihara, Kanagawa 252-5201, Japan; 2 Department of Neurology, Juntendo University School of Medicine, 2-1-1 Hongo, Bunkyo, Tokyo 113-8421, Japan; davide@juntendo.ac.jp; 3 Department of Human Health Care, Tohto College of Health Sciences, 4-2-11 Kamishiba-cho Nishi, Fukaya, Saitama 366-0052, Japan; eiichimomotani@gmail.com

**Keywords:** Cry j 1, Cry j 2, dogs, IgE antibody, Japan

## Abstract

Levels of Japanese cedar pollen (*Cryptomeria japonica*) have increased in Japan and cedar pollinosis caused by Japanese cedar pollen has been reported in dogs. Serum levels of immunoglobulin E (IgE) against Cry j 1 and Cry j 2 in dogs raised in institutes and treated at veterinary hospitals in Japan were thus investigated. A total of 71 sera obtained from two institutes and 87 sera obtained from veterinary hospitals in the Hyogo and Kanagawa Prefectures were analyzed in this study. Serum levels of IgE were measured using the enzyme-linked immunosorbent assay with commercial purified Cry j 1 and Cry j 2. IgE against Cry j 1 and Cry j 2 in sera obtained from the two institutes were detected, despite the dogs being bred in enclosed areas. Moreover, significant differences were noted in the serum levels of IgE against Cry j 1 and Cry j 2 between the two institutes. The number of samples showing Cry j 1 or Cry j 2 levels above the cut-off values was greater in the Kanagawa Prefecture than in the Hyogo Prefecture. In total, 14 dogs showed Cry j 1 and Cry j 2 levels greater than the cut-off values in the Hyogo Prefecture, and only three such dogs were seen in the Kanagawa Prefecture. A significant correlation between serum levels against both allergens was observed (r^2^ = 0.6931, *p* < 0.0001).

## 1. Introduction

Numerous Japanese cedar (*Cryptomeria japonica*) trees were planted about 70 years ago, and the large number of trees aged 30 years or more has led to increased levels of cedar pollen in Japan [1,2]. In humans, the incidence of seasonal allergic rhinitis and pollinosis in response to Japanese cedar pollen has also increased [3,4,5,6]. Japanese cedar pollinosis is a type I allergy and an immunoglobulin E (IgE)-mediated disease [6,7]. The prevalence of allergic rhinitis and cedar pollinosis caused by Japanese cedar pollen is reported to be 23.4% and 26.5%, respectively [6]. The symptoms of these conditions have reduced the quality of life in Japanese people [8].

Pollinosis caused by Japanese cedar pollen has been reported in dogs, and the incidence of atopic dermatitis in dogs sensitized to Japanese cedar pollen is reported to be about 10% [9,10,11,12,13]. Moreover, specific IgE against crude Japanese cedar pollen has been detected in about 20% of atopic dogs in Japan [8]. Thus, allergies to Japanese cedar pollen in dogs are expected at a similar rate as in humans. Cry j 1 and Cry j 2 were identified as the principal allergens in Japanese cedar pollen in 1983 and 1990, respectively [14,15]. However, there have been few investigations to clarify the Japanese cedar pollen-specific IgE retention ratio against Cry j 1 and Cry j 2 in dogs bred in Japan. There have also been few reports on the prevalence of the IgE antibody against Cry j 2 in dogs. The serum levels of IgE antibodies against Cry j 1 and Cry j 2 in dogs housed by breeders and treated at veterinary hospitals in Japan were measured. The aim of this study was to assess the seroprevalence of IgE antibodies against Cry j 1 or Cry j 2 in dogs raised in Japan.

## 2. Materials and Methods

### 2.1. Sera

**Figure 1:**
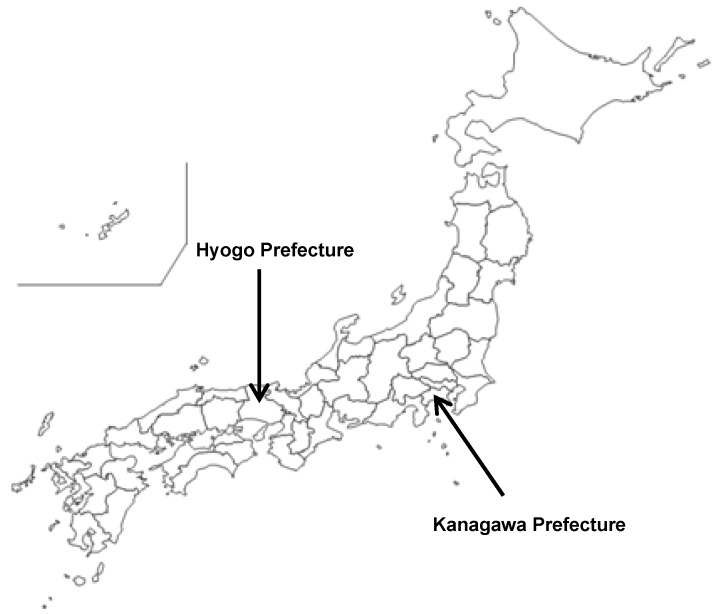
Location of Hyogo Prefecture and Kanagawa Prefecture, Japan.

A total of 71 sera were obtained from beagle dogs raised at three institutes in Japan. Moreover, 87 sera were obtained from various types of dogs treated at veterinary hospitals in Hyogo Prefecture or Kanagawa Prefecture (Figure 1) [16]. Our organization approved the purchase of the sera from the two institutes and veterinary hospitals in Hyogo Prefecture or Kanagawa Prefecture.

### 2.2. Enzyme-Linked Immunosorbent Assay

Serum levels of IgE against Cry j 1 and Cry j 2 were measured using the enzyme-linked immunosorbent assay (ELISA). Purified Cry j 1 or Cry j 2 (Funakoshi Co., Ltd., Tokyo, Japan) was adsorbed onto Immuno 96 microwell^TM^ plates (Thermo Fisher Scientific Inc., Waltham, MA, USA) by incubating 100 µL in 0.05 M sodium bicarbonate buffer (pH 9.6) in each well at 37 °C for 1 h. After blocking with 300 µL/well 20% Blocking One^TM^ (Nacalai Tesque, Inc., Kyoto, Japan) in phosphate-buffered saline (PBS, pH 7.2), ELISA plates were incubated at 37 °C for 1 h and rinsed with PBS (pH 7.2) containing 0.1% polyoxyethylene (20) sorbitan monolaurate (Wako Pure Chemical Industries, Ltd., Osaka, Japan). Sera were added at 100 µL/well. Plates were incubated at 37 °C for 1 h and were rinsed as described previously. Horseradish peroxidase conjugated anti-dog IgE antibody was added at 100 µL/well. After incubation for 1 h, plates were rinsed as described previously. Substrate (1% 2, 2-azino-di-(3-ethyl-benzthiazoline sulphonic acid-6) (ABTS)) was added at 100 µL/well (F. Hoffmann-La Roche Ltd., Basel, Switzerland), and absorbance at 415 nm was measured using a microplate reader (Corona Electric Co., Ltd., Ibaraki, Japan).

### 2.3. Statistics

Data were analyzed using GraphPad Prism 7.0 software (La Jolla, CA, USA). All values are expressed as means ± standard deviation. Serum levels of IgE against Cry j 1 or Cry j 2 were assessed using the unpaired Student’s *t*-test. Cut-off values for serum levels of IgE against Cry j 1 and Cry j 2 were determined using a receiver operating characteristic (ROC) curve analysis of data from two institutes. *p*-values of <0.05 was considered to represent a significant difference.

## 3. Results

**Figure 2:**
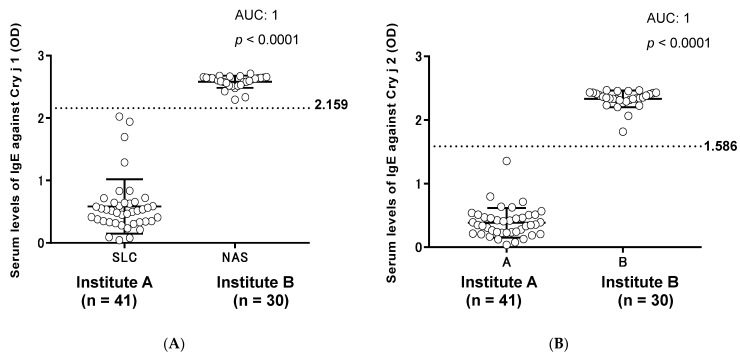
Evaluation of seroprevalence of immunoglobulin E (IgE) antibodies against Japanese cedar pollen allergens Cry j 1 and Cry j 2 in dogs. (**A**) Dot plot showing the distribution of IgE against Cry j 1 in dog serum obtained from Institutes A (n = 41) and B (n = 30) using the enzyme-linked immunosorbent assay (ELISA). (**B**) Dot plot showing the distribution of IgE against Cry j 2 in dog serum obtained from Institutes A and B. The dotted line indicates the cut-off value, while bars indicate means ± standard deviation. AUC, area under receiver operating characteristic (ROC) curve.

**Figure 3:**
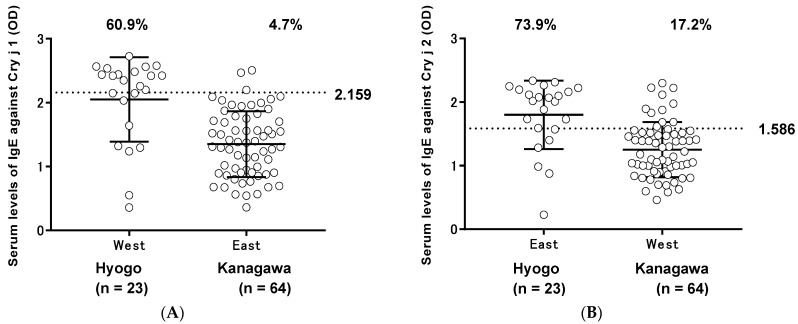
Evaluation of seroprevalence of immunoglobulin E (IgE) antibodies against Japanese cedar pollen allergens Cry j 1 and Cry j 2 in dogs. (**A**) Dot plot showing the distribution of IgE against Cry j 1 in dog serum obtained from veterinary hospitals in Hyogo Prefecture (n = 23) and Kanagawa Prefecture (n = 64) by ELISA. (**B**) Dot plot showing the distribution of IgE against Cry j 2 in dog serum obtained from veterinary hospitals in Hyogo Prefecture and Kanagawa Prefecture. The dotted line indicates the cut-off value, while bars indicate means ± standard deviation. Percentages indicate the number of dogs showing Cry j 1 or Cry j 2 levels above the cut-off values.

**Figure 4:**
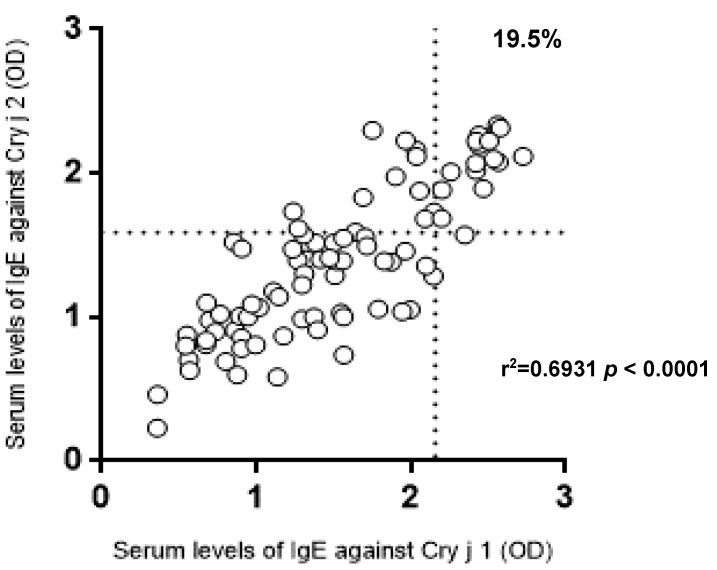
Correlation between serum levels of immunoglobulin E (IgE) against Cry j 1 and against Cry j 2. Sera were obtained from veterinary hospitals in Hyogo Prefecture (n = 23) and Kanagawa Prefecture (n = 64). The dotted lines show the cut-off values. Percentages indicate the number of dogs showing Cry j 1 and Cry j 2 levels above the cut-off values.

The serum levels of IgE against Cry j 1 and Cry j 2 at the two institutes are shown in Figure 1. The serum levels of IgE antibodies against Cry j 1 and Cry j 2 at Institute A were significantly lower than those at Institute B. The cut-off values for the serum levels of IgE against Cry j 1 and Cry j 2 were 2.159 and 1.586, respectively. The serum levels of IgE against Cry j 1 and Cry j 2 obtained from Hyogo Prefecture and Kanagawa Prefecture are shown in Figure 2. At the established cut-off of 2.159, Cry j 1 antigens gave strong ELISA titer values in three out of 64 (4.7%) samples from Kanagawa Prefecture and in 14 out of 23 (60.9%) samples from Hyogo Prefecture. At the established cut-off of 1.586, Cry j 2 antigens gave strong ELISA titer values in 11 out of 64 (17.2%) samples from Kanagawa Prefecture and in 17 out of 23 (73.9%) samples from Hyogo Prefecture. The correlation between the serum levels of IgE against Cry j 1 and Cry j 2 is shown in Figure 3. A significant correlation between serum levels against both allergens was observed in Figure 4 (r^2^ = 0.6931, *p* < 0.0001). In total, 17 of 87 (19.5%) dogs showed Cry j 1 and Cry j 2 levels greater than the cut-off values.

## 4. Discussion

The serum levels of IgE against Cry j 1 and Cry j 2 were estimated in order to clarify the seroprevalence of IgE antibodies against these allergens in dogs in Japan. Essentially, dogs bred in closed rooms at Institutes A and B should not be exposed to Japanese cedar pollen. However, high serum levels of IgE against these allergens were observed at Institute B. Japanese cedar pollen was detected in closed rooms, as it is impossible to completely capture airborne Japanese cedar pollen with standard air conditioner filters [17,18,19,20,21]. Furthermore, air cleaners for homes are unable to eliminate Japanese cedar pollen, and there are differences in effectiveness among various products [21]. The reason for IgE antibodies against Cry j 1 or Cry j 2 being present in dogs raised in enclosed areas was considered to be continuous exposure to Japanese cedar pollen through the air filter. The serum levels of IgE against Cry j 1 and Cry j 2 at Institute A were significantly lower than at Institute B. This significant difference was presumed to be due to differences in the air conditioner filters of these institutes.

The cut-off values for Cry j 1 and Cry j 2 were calculated using a ROC curve analysis of the data from Institutes A and B. The number of samples showing Cry j 1 or Cry j 2 values above the cut-off values were greater in Kanagawa Prefecture than in Hyogo Prefecture. A total of 13 dogs showed Cry j 1 and Cry j 2 levels above the cut-off values in Kanagawa Prefecture, but only three such dogs were seen in Hyogo Prefecture. Regional differences between Hyogo Prefecture and Kanagawa Prefecture in serum levels of IgE against Cry j 1 and Cry j 2 in dogs were Falso observed. Regional differences in the prevalence of the IgE antibody against Japanese cedar pollen have also been observed in humans [22]. Japanese cedar forests in Kochi Prefecture cover more area, and the region thus shows a higher prevalence of cedar pollinosis [8]. The prevalence of cedar pollinosis is considered to be correlated with the forest area of Japanese cedar and the resulting dispersal amount of cedar pollen [8]. The forest area and proportion of forested land in Hyogo Prefecture are larger than those in Kanagawa Prefecture [8]. Airborne levels of Japanese cedar pollen in Hyogo Prefecture were therefore presumed to be higher than in Kanagawa Prefecture, thus leading to higher serum levels of IgE in dogs from Hyogo Prefecture. Sera samples collected in this study were not necessarily obtained from dogs showing any allergic symptoms. No clinical signs in any of the dogs under observation indicated high titers of IgE against Japanese cedar pollen. This suggests that some other factors lead to the development of clinical signs [13]. IgE antibodies against *Dermatophagoides farina* and *Dermatophagoides pteronyssinus* have been found in normal dogs [23]. The fact that dogs without clinical symptoms of allergies possessed IgE antibodies against Cry j 1 and Cry j 2 was therefore not considered to be unusual. Some samples from dogs showing clinical signs of allergies were also collected, but further study is necessary to compare serum levels of IgE against Cry j 1 and Cry j 2 between dogs showing allergic symptoms and those without any allergic symptoms.

A significant correlation between serum levels of Cry j 1 and Cry j 2 was observed. Dogs were considered to be exposed to Cry j 2 at similar levels as Cry j 1. Serum levels of IgE against Cry j 2 were significantly lower than those against Cry j 1, similar to in humans [22]. The quantity of Cry j 2 in Japanese cedar varied each year, while the concentration of Cry j 1 was consistent [24,25,26]. Furthermore, the IgE-binding capacity of Cry j 1 is higher than that of Cry j 2 [25,26]. However, the number of clinical samples in dogs showing Cry j 2 levels above the cut-off values was greater when compared with Cry j 1. Further study is needed in order to clarify the differences in antigenicity between Cry j 1 and Cry j 2 in dogs. It has been reported that major allergens for Japanese cedar pollen and Japanese cypress pollen have a common antigenicity with high homogeneity [27]. Amino acid sequences for Cry j 1 and Cha o 1 have a reported homogeneity of 80%, and those of Cry j 2 and Cha o 2 have been reported at 74% [27]. Further study is needed to examine the cross-reactivity for these allergens.

## 5. Conclusions

The IgE antibodies against Cry j 2 were detected at similar rates as Cry j 1 in dogs raised in Japan. Dogs have IgE antibodies against Cry j 1 and Cry j 2 due to continuous Japanese cedar pollen exposure through air conditioner filters, despite being bred in enclosed areas. The number of samples showing Cry j 1 or Cry j 2 levels above the cut-off values were greater in Kanagawa Prefecture than in Hyogo Prefecture. Regional differences in the prevalence of IgE antibodies in dogs are similar to those seen in humans.
