# Erratum: Kuribayashi, T. et al. Seroprevalence of Immunoglobulin E Antibodies against Japanese Cedar Pollen Allergens Cry j1 and Cry j2 in Dogs Bred in Japan. *Vet. Sci.* 2018, *5*, 79

**DOI:** 10.3390/vetsci5040097

**Published:** 2018-11-30

**Authors:** Takashi Kuribayashi, Davide Cossu, Eiichi Momotani

**Affiliations:** 1 Laboratory of Immunology, School of Life and Environmental Science, Azabu University, Fuchinobe 1-17-71, Chuo-ku, Sagamihara, Kanagawa 252-5201, Japan; 2 Department of Neurology, Juntendo University School of Medicine, 2-1-1 Hongo, Bunkyo, Tokyo 113-8421, Japan; davide@juntendo.ac.jp; 3 Department of Human Health Care, Tohto College of Health Sciences, 4-2-11 Kamishiba-cho Nishi, Fukaya, Saitama 366-0052, Japan; eiichimomotani@gmail.com

Due to an error during production, the column title of Figure 2 and Figure 3 are misaligned in the Results section of the published paper [1]. A corrected version of the Figures and associated captions are provided below. Please note that these alterations do not modify the primary data, their significance or the related conclusions. The authors apologize for any inconvenience caused by this minor error. The manuscript will be updated and the original will remain online on the article webpage.

## Figures and Tables

**Figure 2:**
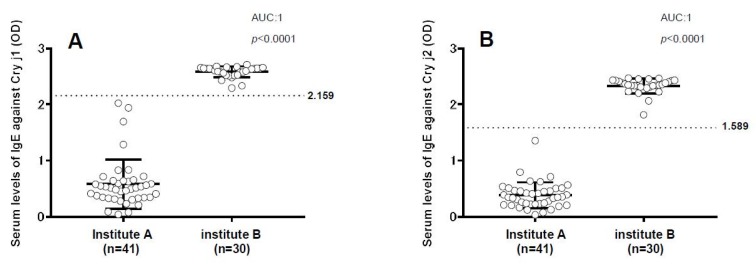
Evaluation of seroprevalence of immunoglobulin E (IgE) antibodies against Japanese cedar pollen allergens Cry j1 and Cry j2 in dogs (**A**) Dot plot showing the distribution of IgE against Cry j1 in dog serum obtained from institutes A (n = 41) and B (n = 30) by enzyme linked immunosorbent assay (ELISA). (**B**) Dot plot showing the distribution of IgE against Cry j2 in dog serum obtained from institutes A and B by ELISA. The dotted line indicates the cut-off value, while bars indicate the mean ± standard deviation.

**Figure 3:**
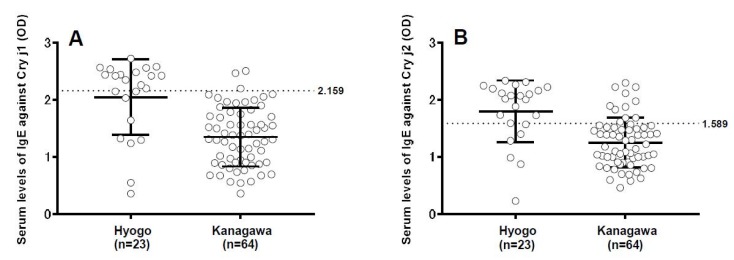
Evaluation of seroprevalence of immunoglobulin E (IgE) antibodies against Japanese cedar pollen allergens Cry j1 and Cry j2 in dogs (**A**) Dot plot showing the distribution of IgE against Cry j1 in dog serum obtained from veterinary hospitals in Hyougo Prefecture (n = 23) and Kanagawa Prefecture (n = 64) by ELISA. (**B**) Dot plot showing the distribution of IgE against Cry j2 in dog serum obtained from veterinary hospitals in Hyougo Prefecture and Kanagawa Prefecture by ELISA. The dotted line indicates the cut-off value, while bars indicate the mean ± standard deviation.

## References

[1] Kuribayashi T., Cossu D., Momotani E. (2018). Seroprevalence of Immunoglobulin E Antibodies against Japanese Cedar Pollen Allergens Cry j 1 and Cry j 2 in Dogs Bred in Japan. Vet. Sci..

